# Disability Affects the 6-Minute Walking Distance in Obese Subjects (BMI>40 kg/m^2^)

**DOI:** 10.1371/journal.pone.0075491

**Published:** 2013-10-11

**Authors:** Lorenzo Maria Donini, Eleonora Poggiogalle, Veronica Mosca, Alessandro Pinto, Amelia Brunani, Paolo Capodaglio

**Affiliations:** 1 Department of Experimental Medicine–Medical Physiopathology, Food Science and Endocrinology Section, “Sapienza” University of Rome, Rome, Italy; 2 Rehabilitation Unit and Research Laboratory in Biomechanics and Rehabilitation, Istituto Auxologico Italiano IRCCS, Piancavallo, Verbania, Italy; University of Sao Paulo, Brazil

## Abstract

**Introduction:**

In obese subjects, the relative reduction of the skeletal muscle strength, the reduced cardio-pulmonary capacity and tolerance to effort, the higher metabolic costs and, therefore, the increased inefficiency of gait together with the increased prevalence of co-morbid conditions might interfere with walking. Performance tests, such as the six-minute walking test (6MWT), can unveil the limitations in cardio-respiratory and motor functions underlying the obesity-related disability. Therefore the aims of the present study were: to explore the determinants of the 6-minute walking distance (6MWD) and to investigate the predictors of interruption of the walk test in obese subjects.

**Methods:**

Obese patients [body mass index (BMI)>40 kg/m^2^] were recruited from January 2009 to December 2011. Anthropometry, body composition, specific questionnaire for Obesity-related Disabilities (TSD-OC test), fitness status and 6MWT data were evaluated. The correlation between the 6MWD and the potential independent variables (anthropometric parameters, body composition, muscle strength, flexibility and disability) were analysed. The variables which were singularly correlated with the response variable were included in a multivariated regression model. Finally, the correlation between nutritional and functional parameters and test interruption was investigated.

**Results:**

354 subjects (87 males, mean age 48.5±14 years, 267 females, mean age 49.8±15 years) were enrolled in the study. Age, weight, height, BMI, fat mass and fat free mass indexes, handgrip strength and disability were significantly correlated with the 6MWD and considered in the multivariate analysis. The determination coefficient of the regression analysis ranged from 0.21 to 0.47 for the different models. Body weight, BMI, waist circumference, TSD-OC test score and flexibility were found to be predictors of the 6MWT interruption.

**Discussion:**

The present study demonstrated the impact of disability in obese subjects, together with age, anthropometric data, body composition and strength, on the 6-minute walking distance.

## Introduction

In obese subjects, the relative reduction of the skeletal muscle strength [1], the reduced cardio-pulmonary capacity and tolerance to effort [2], [3], the higher metabolic costs and, therefore, the increased inefficiency of gait [4], together with the increased prevalence of co-morbid conditions, might interfere with walking. Pain from overloaded joints [5]–[7] is a frequent complaint during walking in obese subjects, who tend to walk slower and report more frequently dyspnea than their lean counterparts [8]. On the other hand, walking often represents the most accessible mean of exercise for weight management. The ability to walk for a distance is a quick and inexpensive measure of physical function, and an important component of quality of life, since it reflects the capacity to undertake the activities of daily living [4], [5]. Performance tests, such as the six-minute walking test (6MWT), can unveil the limitations in cardio-respiratory and motor functions underlying the obesity-related disability [2], [3].

After the publication of the 6MWT official guidelines elaborated by the American Thoracic Society in 2002, several authors studied the determinants of the 6-minute walking distance (6MWD) in healthy adults. Predictive equations considering age, sex, weight and height were proposed for clinical use [9]–[13]. They aimed at representing a reference test for populations with different ethnicities and clinical conditions. These studies varied with respect to the number of individuals (with the exception of two large ones) [14], [15] but presented similar design and the reference equations were obtained using linear multiple regression models, including demographic and anthropometric features (age, sex, stature and weight in almost all studies) [16]. Only few studies correlated the 6MWD and severity of obesity; moreover, despite results were shown to be highly reproducible, they also demonstrated that they were influenced by the severity of obesity, reduced strength and aerobic capacity [17], [18].

According to the predictive equations from the literature, obese subjects consistently show a deficit in the distance walked and in work exerted for walking when compared with normal-weight subjects [19]. Reference values obtained from healthy, normal-weight populations would therefore predictably underline the reduced performance capacity of obese individuals. Instead, reference values specific for this population would serve as a benchmark to assess baseline functional capacity, to prescribe proper and safe exercise intensity and to supervise changes after rehabilitation interventions. Recently Capodaglio et al. [18] developed a reference equation for predicting the 6MWD specifically in adult obese subjects to be used in the clinical practice. Clinical applicability of the test represented, for many authors, the guiding criterium for avoiding inclusion of other parameters correlated with the results of the walking test. From a mathematical point of view, the correlation with the 6MWD would certainly benefit from the inclusion of several other factors in the predictive formula. Hulens et al. [8] found that 75% of the variance in walking performance was explained by the combination of the following variables: body mass index (BMI), peak aerobic capacity, knee extension torque, age, hours of TV viewing, BMI explaining 59% of the variance by itself. Among the predictors of the distance walked, other physiological (heart rate, oxygen saturation, blood pressure, muscle strength), life style (physical activity levels) factors and degree of disability may well play a role. Although their inclusion in an equation appears unpractical for clinical use, we need to further investigate the determinants of distance walked by obese individuals, as it would result likely in an increased prediction capacity of the equation and a deeper comprehension of the limitations of obese subjects. Also, pre- and post-assessments after combined interventions in obese subjects revolve around the main expected outcome of weight loss. The expected functional correlation is an increase in the distance walked secondary to weight loss. However, if co-morbid disabling conditions are present, distance might not necessarily increase, as expected on the basis of weight loss solely. Otherwise, if weight loss is accompanied by an improved tolerance to the effort after aerobic conditioning, the formula may underestimate the real performance. Hence, we hypothesized that the degree of disability of obese subjects should be part of their functional assessment. In fact, their disability was shown to affect the basic activities of daily living and to be mainly related to mobility impairment. Recently, an obesity-specific disability scale was developed [20] and it was also demonstrated to be able in measuring changes after multidisciplinary rehabilitation interventions [21], [22]. Therefore, the aims of the present study were: to further explore the determinants of the 6MWD by obese subjects and in particular whether measures of disability would affect the results; and to investigate the predictors of interruption of the walk test in obese subjects.

## Methods

### Subjects

Caucasian adult obese patients (BMI>40 kg/m^2^) were recruited at the Metabolic, Nutritional and Psychological Rehabilitation Unit at “Villa delle Querce” Clinical and Rehabilitation Institute (Nemi, Rome-Italy) from January 2009 to December 2011, among all the obese patients hospitalised in the facility during the above mentioned period. Eligibility criteria for patients to be admitted to an intensive rehabilitation treatment were: BMI>40 kg/m^2^ associated to a significant disability level [as assessed by the TSD-OC test (SIO Test assessing disabilities obesity related), see above, with a disability score>33% - [20] and the presence of at least one clinical comorbidity. Patients aged less than 18 years and more than 80 years were excluded from the study. In addition, bed-ridden patients and patients presenting contraindications for the 6MWT (acute cardiac diseases in the previous month, unstable angina, uncontrolled hypertension (higher than 180/100 mmHg), major othopaedic or neurological conditions interfering with the test) were excluded [23].

The study protocol was approved by the Ethical Committee of the “Sapienza” University of Rome and oral and written informed consent was obtained from all the subjects.

### Measurements

The following data were measured within the first week after the admission:

- anthropometric measures, according to the procedures described in the “Anthropometric standardisation reference manual” by Lohman et al. [24], by a trained operator. Body weight was measured to the nearest 0.1 kg using a standard column body scale SECA (Hamburg, Germany). Body height (using a rigid stadiometer – SECA, Hamburg, Germany), waist and arm circumferences (WC and AC respectively) (using a measuring tape) were determined to the nearest 0.1 cm. Triceps skinfold thickness (TSF) was measured using a Harpenden Skinfold Caliper (British Indicators Ltd, St. Albans, Herts, UK).

Then, the following indexes were calculated:

-
  - BMI =  weight/height in kg/m^2^
  - mid-upper arm muscle circumference  = AC - (π * TSF)
- Body composition [fat mass (FM) and fat free mass (FFM)] was estimated by bioelectrical impedance analysis (BIA): whole-body impedance vector components, resistance (R) and reactance (Xc), were measured with a single-frequency 50-kHz analyzer STA-BIA (AKERN Bioresearch SRL, Pontassieve, FL, Italy). Measurements were obtained following standardized procedures [25]. The external calibration of the instrument was checked with a calibration circuit of known impedance value. Estimations of FFM and FM by BIA were obtained using sex-specific, BIA prediction equations developed by Sun et al. in a large population including extremes of BMI values [26]. Fat mass index (FMI) and fat-free mass index (FFMI) were calculated as FM or FFM in kg/body height in m^2^.
- Specific short-form questionnaire for Obesity-related Disabilities (TSD-OC test) proposed by the Italian Society of Obesity was fulfilled by all the participants [20]. The TSD-OC test addresses adults and does not target a specific sex. It is composed by 7 sections (pain: 5 items; stiffness: 2 items; activities of daily living and indoor mobility: 7 items; housework: 7 items; outdoor activities: 5 items; occupational activities: 4 items; social life: 6 items) for a total of 36 items. Patients were requested to subjectively assess their difficulty in each item by means of a 0–10 visual analogue scale (10 indicating the highest level of disability and 0 no difficulties in performing the task). The total score (0 to 360) represents the disability status of the patient;
- Fitness status was assessed by:
  - hand grip strength (HGST), measured using a Lafayette hand grip (Mod. 78011). The maximum value (kg) out of three trials using the dominant hand was recorded. Between two consecutive trials, a 1-minute recovery was provided [27];
  - Spine flexion, together with hip and shoulder flexion, extension, and abduction were measured with a standard goniometer by a skilled physiotherapist. The floor-fingertip distance (in centimeters) was considered as a measure of spinal flexibility;
- The 6MWT was performed according to the instructions by the American Thoracic Society [23]. In particular, conditions for the execution of a safe test were respected: an easily accessible corridor for emergencies, the test interruption criteria, such as chest pain, severe dyspnea, muscle cramps, dizziness, and sudden paleness, were considered when applicable. The test was performed in an undisturbed 20-meter hospital corridor marked every 2 meters with colored tape on the floor; starting and finishing points were marked on the floor. Before the test, at 1, 3 and 5 minutes after the start and at the end of the test, pulse, respiratory rate, blood pressure and perceived fatigue on Borg's scale were measured [28]. Subjects were instructed to walk as fast as they could. They were allowed to stop or rest during the test if necessary. The 6MWD was calculated.

### Statistics

First, the correlations between 6MWD and the potential independent variables (anthropometric parameters, body composition, muscle strength, flexibility and disability) were analysed. After verification of the normal distribution of the variables, t-test and the analysis of variance (ANOVA) were performed to describe differences between means of the groups, and chi-square test was used to compare observed and expected frequencies. A linear regression analysis (Pearson's r) was performed to verify the association among continuous variables.

In a second phase, the variables which were singularly correlated with the response variable were included in a group of potential explicative elements of a multivariated regression model using the variables with the highest correlations and excluding the redundant ones to minimize the confounding effect of collinearity, in accordance with the principle of parsimony.

The multiple linear regression models obtained were expressed in the following algebraic form
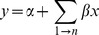
where “y” represents the outcome variable (6MWD), “x” the values of the independent variables, “β” the unstandardized coefficients of the independent variable and α the constant intercept coefficient.

The efficacy of the regression model was analysed according to the value of the determination coefficient R^2^ (comparing the explained variance of the model's predictions with the total variance of the data) and the R^2^adjusted (considering a correction for inclusion of variables). The standard error of the estimate (SEE), representing a measure of the accuracy of predictions (standard deviation of the differences between the actual values of the dependent variables (results) and the predicted values), was calculated.

Finally, the correlations between nutritional and functional parameters and test interruption were investigated.

Differences were considered to be statistically significant at p<0.05. Statistical analysis was performed using SPSS 10.0 statistical software (SPSS Inc Wacker Drive, Chicago, IL, USA).

## Results

### Characteristics of the study sample (Table 1)

354 subjects (87 males, mean age 48.5±14 years - range 19–74 years, 267 females, mean age 49.8±15 years - range 19–80 years) were enrolled in the study. All of the subjects had a BMI>40 kg/m^2^ (44.7±8 versus 43.7±8 kg/m^2^, respectively for males and females) with a significantly increased WC (133.3±13 versus 117.8±15 cm, respectively for males and females; p<0.05). Statistically significant differences (p<0.05) were found between males and females, in particular for the 6MWD (444.3±106 versus 418.8±80 m), handgrip strength (36.7±7 versus 25.4±6 kg) and articular mobility.

### Deteminants of the 6MWD

In Table 2, the correlations between the considered variables and the distance walked are described. Based on these results, a multivariate regression analysis was performed using only the independent variables significantly correlated with the outcome variables at the univariate analysis: age, weight, height, BMI, FMI, FFMI, HGST and disability (TSD-OC test score). Variables showing a lower correlation with analogous biological meaning were excluded. Sex was not part of the predictive model: distance walked by males and females did not significantly differ in our sample (Table 1). Data from the elaborated models and indicators of the precision in describing the 6MWT results are reported in Table 3. The R^2^ of the regression analysis ranged from 0.21 of the model 1 considering only HGST and TSD-OC (SEE: 82.0 m) to 0.47 for the model 5 considering also age, FMI and FFMI (SEE: 66.7 m). Slightly lower results were obtained with models using BMI or body weight and height. Model 5 showed a significant correlation with the real distance walked by patients (r = 0.644; p<0.001): the mean difference between real and predicted results was 38.7±79 m (range −42.5 m to 106.1 m).

**Table 1:** Demographic and functional characteristics of the entire sample (n = 354).

		Males	Females	p<0.05
**N**		87	267	
**Age (years)**		48.5±14	49.8±15	
**Anthropometry and body composition**	**Weight (kg)**	134.1±22	111.1±21	*
	**BMI (kg/m^2^)**	44.7±8	43.7±8	
	**TSF (mm)**	31.5±11	38.9±9	*
	**AC (cm)**	39.3±4	41.4±5	
	**MAMC (cm)**	29.6±4	29.2±5	
	**WC (cm)**	133.3±13	117.8±15	*
	**FMI (kg/m^2^)**	18.9±3	23.2±3	*
	**FFMI (kg/m^2^)**	26.0±4	20.8±3	*
**Function**	**6MWD (m)** §	444.3±106	418.8±80	
	**Hand grip strength (kg)**	37.9±7	26.8±6	*
**Articular mobility**	**Spine flexion (cm)**	17.7±10	11.4±11	*
	**Hip flexion - right (°)**	76.2±17	79.1±18	
	**Hip flexion – left (°)**	75.7±16	80.0±15	*
	**Hip extension - right (°)**	22.7±7	20.0±7	*
	**Hip extension – left (°)**	23.1±7	20.0±7	*
	**Hip abduction - right (°)**	36.5±7	36,1±8	
	**Hip abduction - left (°)**	37.3±11	37.4±11	
**Disability**	**TSD-OC (%)**	33.2±26	36.8±25	

§The data refer only to persons who have completed the 6MWT (M:74, F:221)

*p<0.05: t test: males versus females

***L*** *egend*: BMI: body mass index; TSF: triceps skinfold thickness; AC: arm circumference; MAMC: mid-upper arm muscle circumference; WC: waist circumference; FMI: fat mass index: FFMI: fat-free mass index; 6MWD: six-minute walking distance; TSD-OC: specific short-form questionnaire for Obesity-related Disabilities proposed by the Italian Society of Obesity.

**Table 2:** Correlation between 6-minutes walking distance and functional - nutritional parameters.

		*Correlation coefficient*	*p*
**Age**		−0.37	0.000
**Antropometry and body composition**	Weight	−0.25	0.000
	Height	0.35	0.001
	BMI	−0.39	0.000
	WC	−0.21	NS
	AC	−0.34	0.000
	MAMC	−0.28	0.000
	TSF	−0.07	NS
	FMI	−0.45	0.005
	FFMI	0.21	NS
**Strength**	HGST	0.36	0.000
**Articular Mobility**	Spine flexion	−0.24	0.000
	Hip flexion - right	−0.25	0.000
	Hip flexion – left	−0.29	0.000
	Hip extension - right	−0.18	0.001
	Hip extension – left	−0.15	0.005
	Hip abduction - right	−0.13	0.016
	Hip abduction - left	−0.18	0.005
**Disability**	TSD-OC	−0.36	0.000

The data refer only to persons who have completed the six-minute walk test (M:74, F:221)

***L*** *egend*: BMI: body mass index; WC: waist circumference; AC: arm circumference; MAMC: mid-upper arm muscle circumference; TSF: triceps skinfold thickness; FMI: fat mass index; FFMI: fat-free mass index; HGST: hand-grip strength test; TSD-OC: specific short-form questionnaire for Obesity-related Disabilities proposed by the Italian Society of Obesity.

**Table 3:** Multivariate model correlating 6-minute walk distance (6MWD) to clinical and functional parameters.

	Model 1			Model 2			Model 3			Model 4			Model 5		
	β	SE (β)	p	β	SE (β)	p	β	SE (β)	p	β	SE (β)	p	β	SE (β)	p
HGST	3.11	0.2	0.000	2.14	0.54	0.000	1.73	0.66	0.008	2.73	0.49	0.000	3.37	0.95	0.000
TSD-OC	−1.13	0.54	0.000	−0.97	0.2	0.000	−0.88	0.19	0.000	−0.56	0.18	0.002	−0.47	0.25	0.007
Age				−1.85	0.32	0.000	−1.59	0.31	0.000	−1.77	0.29	0.000	−1.30	0.46	0.005
Weight							−1.23	0.22	0.000						
Stature							3.03	0.59	0.000						
BMI										−4.87	0.57	0.000			
FMI													−5.69	1.27	0.000
FFMI													1.47	0.11	0.002
Intercept	382.17	18.6	0.000	496.61	26.9	0.000	67.11	108.0	0.524	670.59	31.9	0.000	577.82	48.1	0.000
**R^2^**	0.21			0.28			0.38			0.41			0.47		
**R^2^ adj**	0.21			0.28			0.37			0.41			0.45		
**SEE**	82.0			77.5			72.8			70.5			66.9		

***Legend***: HGST: hand grip strength test; TSD-OC: specific short-form questionnaire for Obesity-related Disabilities; BMI: body mass index; FMI: fat mass index; FFMI: fat-free mass index; β: the unstandardized coefficients of the independent variable; R^2^: determination coefficient; SE: standard error; SEE: standard error of the estimate.

Example of regression equation: Model 1: 6MWD (m)  = 382.17+(3,11 * HGST) − (1.13 * TSD-OC).

### Predictors of the 6MWT interruption (Table 4)

**Table 4:** Nutritional and functional parameters in completers (C) and not completers (NC) the 6MWT.

		Males		Females	
		C	NC	C	NC
**N**		72	15	213	54
**Age (years)**		50.1±13	48.9±14	50.2±13	53.4±14
**Anthropometry and body composition**	**Weight (kg)**	131.1±18	144.2±33*	106.8±20	121.3±23*
	**BMI (kg/m^2^)**	44.1±7	49.5±7*	43.4±7	47.7±9*
	**AC (cm)**	38.1±4	42.2±6*	38.9±4	42.4±5*
	**MAMC (cm)**	29.1±3	31.2±5	28.6±4	31.3±5*
	**WC (cm)**	132.6±11	143.5±16*	117.2±14	126.4±17*
	**FMI (kg/m^2^)**	17.9±3	22.0±5*	22±5	25.6±4*
	**FFMI (kg/m^2^)**	25.1±3	26.8±4	20.4±2	21.7±4
**Strength**	**HGST (kg)**	37.3±7	38.4±5	27.4±5	25.6±4*
**Articular mobility**	**Spine flexion (cm)**	18.7±9	15.9±8	10.2±10	14.6±11*
	**Hip flexion - right (°)**	77.4±14	70.0±22*	80.2±16	72.9±19*
	**Hip flexion – left (°)**	76.5±16	73.3±14*	81.8±13	74.2±17*
	**Hip extension - right (°)**	23.7±7	22.5±9*	21.2±7	18.6±7*
	**Hip extension – left (°)**	23.9±6	21.2±8*	22±7	19.2±8*
	**Hip abduction - right (°)**	38.2±6	32.3±6*	37±9	33.9±9*
	**Hip abduction - left (°)**	39.1±11	34.1±6*	38.1±9	32.4±9*
**Disability**	**TSD-OC (%)**	27.4±26	48.7±22*	33.5±24	44.1±28*

* *p<0.05*.

*Legend*: 6MWT: six-minute walk test; BMI: body mass index; AC: arm circumference; MAMC: mid-upper arm muscle circumference; WC: waist circumference; FMI: fat mass index: FFMI: fat-free mass index; HGST: hand grip strength test; TSD-OC: specific short-form questionnaire for Obesity-related Disabilities.

15 males (17.2%) and 54 females (30.2%) interrupted the test according to the described criteria (p>0.05).

Obese men who interrupted the test showed a higher body weight (144.2±33 versus 131.1±18 kg), BMI (49.5±7 versus 44.1±7 kg/m^2^) and WC (143.5±16 versus 132.6±11 cm) (p<0.05) than the rest of the sample. Disability as measured by TSD-OC test was more severe: 48.7±22 versus 27.4±26% (p<0.05). Flexibility, except for spine flexion, were significantly lower (p<0.05). Although non-significantly, among those who interrupted the test, HGST showed a tendency to be lower and FM higher.

Obese women who interrupted the test showed a higher body weight (121.3±23 versus 106.8±20 kg), BMI (47.7±9 versus 43.4±7 kg/m^2^), a larger WC (126.4±17 versus 117.2±14 cm) and higher FM (47.6±4 versus 44.8±4%) than obese women completing the test (p<0.05). The degree of disability was also higher (44.1±28 versus 33.5±24%; p<0.05), whereas HGST (25.4±7 vs 27.2±5 kg) and flexibility were significantly lower (p<0.05). Males and females did not differ significantly with respect to age.

## Discussion

The present study demonstrated the impact of the degree of disability in obese subjects on the 6MWD. The latter was correlated to the following variables: age, anthropometric data (body weight, height, BMI), body composition (FMI, FFMI), strength (HGST) and disability (TSD-OC test).

Previously several authors addressed the identification of determinants of the 6MWD by healthy adults and proposed reference equations. The large majority of them considered only body height, age and body weight [16]. Troosters et al. [10] concluded that these variables accounted for 66% of the variance in a sample of 53 healthy Caucasian adults aged 50 to 85 years, who were not previously hospitalized and did not show any chronic condition potentially hindering physical capacity [29]. Enright [30] performed the 6MWT in 290 healthy adults aged 40 to 80 years with BMI<35 kg/m^2^, finding a significant difference depending on height, sex and age. There is a general consensus about the fact that shorter individuals and females present a shorter step length and, consequently, shorter distances walked at the 6MWT. Likewise, in elderly sarcopenic individuals, similarly to patients with cognitive impairment or musculoskeletal disorders, reduction in the 6MWD was described [14], [30].

Muscle strength, depression, reduced perceived quality of life, medications, inflammatory disease and impaired pulmonary function are other factors that can influence the test performance [31]–[34]. In particular, in a study done by Enright and Sherrill [9], a BMI>30 kg/m^2^ was considered an exclusion criterium, since the research addressed the adult healthy population. Also a paper by Hulens et al. [8] was in line with these considerations, underlining that the test results were highly affected by the degree of obesity. Ben Saad et al. [13] showed that when BMI was included in the final reference equation, the 6MWD decreased by 5.27 meters when BMI increased by one unit. In a later study [30], Enright reported that the 6MWT results were affected by muscle strength in individuals with reduced mobility and aerobic capacity. Thus, our results are consistent with the extant literature: mobility and muscle strength are key factors for predicting the 6MWD by obese individuals. Body composition was considered relevant by some authors in influencing results at the 6MWT, more significantly than BMI per se [18], [30]. Although the BMI is a useful epidemiological index of obesity, it cannot be considered as the best index to determine the amount of body fat. Moreover, the correlation between body composition and the 6MWD is usually more robust than the correlation between the 6MWD and BMI [14], [30], [31], [34]. In our sample, these data were confirmed, both FMI and FFMI, and HGST correlating with the 6MWD. We also aimed at ascertaining to what extent disability may affect test results. In a previous study Enright [30] concluded that disability in activities of daily living and occupational activities is an important factor. Disability may impair the test performance also at the emotional and psychological level, as it may induce depression, which ultimately impacts on the 6MWT results, according to several authors [14], [35]–[37]. In fact, also the American Thoracic Society in the guidelines published in 2002 [23], recommended the use of standardized encouragement to avoid bias of the results, on the basis that improving the emotional state may enhance 6MWD results by 30%. Despite significantly correlated to the distance walked, the proposed multivariate models explained less than half of the variance of the phenomenon. The other models in the literature show R^2^ ranging from 0.20 [14] to 0.78 [37]. The population considered in our study may in part explains the relatively low reliability of the model proposed, despite the inclusion of variables all individually correlated with the outcome variable. In fact our population consisted of subjects admitted to a multidisciplinary metabolic- nutritional rehabilitation due to the severe obesity-related comorbidities. They were in frail functional and clinical conditions. Other variables more focused on the clinical aspects may perhaps increase the validity of the model. Other authors [18], [35]–[38] commented that some features linked to specific comorbidities may affect test results; our data about the subjects who were not able to complete the 6MWT seem to be consistent. In fact, obese subjects who failed in the test performance, showed a greater functional impairment and disability, reduced muscle strength, higher fat mass as compared to their counterparts who finished the test. Therefore, the 6MWT appears more as a global performance test than a mere measure of motor capacity. It remains true that the implementation of those variables hinders the daily use of the predictive equation in non-specialistic facilities. However, those variables should be considered in the baseline assessment of obese patients to optimize the rehabilitation programs and increase their effectiveness. The variables adopted in our model define a more complex equation than those already available in the literature, however, the main goal of our study was not to provide an evaluation tool for everyday practice, instead to highlight the differences in the 6MWT results due to the disability correlated to obesity and define the elements that may account for such different performances, either causes or consequences of disability.

The present study has certain limitations that need to be taken into account. Despite having acknowledged all the indications suggested by the American Thoracic Society, the length of the walkway we used in this study was shorter than that used by Enright (20 versus 30 m) [14]. This difference might have biased the results, although it appears very unlikely, as already commented by other authors [35], that this particular circumstance might have caused such a marked difference in the results.

In our study a greater number of females was enrolled. In the literature, as in our study, males normally walk a longer 6MWD. Although the distribution of FM, that is different between males and females, may play a role in influencing this result, evidence suggests that the impact of sex on joint mobility does not appear relevant. Accordingly, in our sample, the correlation between disability and 6MWD does not change as a function of sex.

Some parameters that were shown to be correlated with the performance during the 6MWT (such as customary physical activity, smoking habits, socioeconomic status, depression, lower cognition) [16] were not considered in our study. Although important, however, these aspects were beyond our goals.

Finally, we did not consider in our study the relationship between the 6MWD and parity, an interesting factor in developing nations (4.3 in North Africa and 1.6 in Europe and North America). It seems that parity accelerates decline of the 6MWD [13]. Although in our sample only Caucasian subjects were enrolled, as in Italy there is a large number of immigrant, this association should be evaluated in future studies.

In conclusion, the 6MWD by obese subjects is not only influenced by age, sex and height, as reported in the majority of reference equations in the extant literature. Disability should be a pivotal variable of the predictive model of the distance walked by obese subjects at the 6MWT.
